# β1 Integrin-Mediated Adhesion Signalling Is Essential for Epidermal Progenitor Cell Expansion

**DOI:** 10.1371/journal.pone.0005488

**Published:** 2009-05-08

**Authors:** Aleksandra Piwko-Czuchra, Heidi Koegel, Hannelore Meyer, Martina Bauer, Sabine Werner, Cord Brakebusch, Reinhard Fässler

**Affiliations:** 1 Max Planck Institute of Biochemistry, Department of Molecular Medicine, Martinsried, Germany; 2 ETH Zurich, Institute of Cell Biology, Department of Biology, Hönggerberg, Zurich, Switzerland; 3 Biotech Research and Innovation Centre, University of Copenhagen, Copenhagen, Denmark; Dresden University of Technology, Germany

## Abstract

**Background:**

There is a major discrepancy between the in vitro and in vivo results regarding the role of β1 integrins in the maintenance of epidermal stem/progenitor cells. Studies of mice with skin-specific ablation of β1 integrins suggested that epidermis can form and be maintained in their absence, while in vitro data have shown a fundamental role for these adhesion receptors in stem/progenitor cell expansion and differentiation.

**Methodology/Principal Findings:**

To elucidate this discrepancy we generated hypomorphic mice expressing reduced β1 integrin levels on keratinocytes that developed similar, but less severe defects than mice with β1-deficient keratinocytes. Surprisingly we found that upon aging these abnormalities attenuated due to a rapid expansion of cells, which escaped or compensated for the down-regulation of β1 integrin expression. A similar phenomenon was observed in aged mice with a complete, skin-specific ablation of the β1 integrin gene, where cells that escaped Cre-mediated recombination repopulated the mutant skin in a very short time period. The expansion of β1 integrin expressing keratinocytes was even further accelerated in situations of increased keratinocyte proliferation such as wound healing.

**Conclusions/Significance:**

These data demonstrate that expression of β1 integrins is critically important for the expansion of epidermal progenitor cells to maintain epidermal homeostasis.

## Introduction

Integrins are heterodimeric cell surface receptors consisting of one α and one β subunit. They bind extracellular matrix (ECM) proteins and counter receptors such as VCAM, and play fundamental roles for tissue development and homeostasis [1]. The mammalian genome contains 18 α and 8 β integrin genes whose proteins can give rise to 24 different integrin heterodimers. Integrins are expressed on almost all cells including keratinocytes of the skin.

The epidermis is a multilayered epithelium that protects from environmental assault and damage. The hallmark of the epidermis is its ability to self-renew throughout the entire life span of the organism. This is achieved with epidermal progenitor cells (EPCs) in the basal cell layer of the epidermis, which undergo an unlimited number of symmetric and asymmetric cell divisions, giving rise to daughter cells that either proliferate or exit the cell cycle and move to the suprabasal layer of the epidermis. There they undergo terminal differentiation and are eventually shed from the skin surface [2]. Basal keratinocytes of the murine skin normally express high levels of α2β1, α3β1 and α6β4 integrins and ανβ5 that is weakly expressed. Expression of α5β1, ανβ6 and α9β1 integrins is induced upon wounding or in pathological conditions [3]. Tissue specific or constitutive deletion of these integrin chains revealed their crucial roles in skin development and homeostasis with the most striking phenotypes observed upon deletion of the β1 integrins and the hemidesmosomal α6β4 integrins [4]–[7].

Mutant mice lacking β1 integrin in skin had multiple blisters due to impaired attachment of basal keratinocytes to the basement membrane (BM), a disorganized and hyperthickened interfollicular epidermis (IFE), impaired proliferation of interfollicular and hair matrix keratinocytes, delayed terminal differentiation in IFE, defects in the hair follicle morphology, progressive hair loss and dermal fibrosis [6]. Moreover, β1-deficient mice showed severely delayed wound healing, which has been associated with impaired migration of β1 integrin-deficient keratinocytes [8]. Despite the severe skin defects and complete hair loss, some of the β1 skin specific conditional knockout mice survived up to 12 months suggesting that loss of β1 integrin is not associated with a stem cell or progenitor cell depletion phenotype. This finding was unexpected, as previous data pointed to an important role for β1 integrins for maintaining skin stem cells [9]–[11]. It has been shown that keratinocytes with high β1 integrin levels display typical stem cell properties including high colony forming efficiency in vitro. Similar results were also obtained with cells from adult human palm, sole and breast skin that strongly expressed β1 integrins [12]. Furthermore, it was shown that keratinocytes expressing high levels of β1 integrin are slow cycling cells in vivo and cluster in the basal layer of the epidermis. Interestingly, integrin mediated adhesion to the ECM was shown to negatively regulate terminal differentiation of cultured keratinocytes in vitro, while ectopic expression of β1 integrins in suprabasal cells leads to epidermal hyper-proliferation and perturbed keratinocyte differentiation in vivo [13], [14].

In order to clarify the discrepancy between the in vitro and in vivo results on the role of β1 integrin in the maintenance of epidermal stem cells we generated a mouse strain with reduced β1 integrin expression in keratinocytes of the skin. This approach allows to study the function of β1 integrin in skin homeostasis avoiding the gross abnormalities seen in mice with skin specific ablation of β1 integrin. Using this new model we could show that keratinocytes that express normal levels of β1 integrin due to inefficient downregulation of β1 integrin expression are able to quickly expand in the interfollicular epidermis and almost completely replace the mutant cells over time. This rapid expansion of cells ensures nearly normal epidermal homeostasis and strongly indicates that β1 integrins play a very critical role for the proliferation and maintenance of EPC in vivo.

## Results

### Generation of a β1 integrin hypomorphic allele

To test how reduced expression of β1 integrin affects skin development and maintenance we engineered a β1 hypomorphic mouse strain by diminishing the stability of the β1 integrin mRNA and thus the amount of β1 integrin subunits. The hypomorphic gene mutation was obtained by introducing a 141-bp long wild-type (wt) cDNA fragment of the β1 integrin gene coding for the entire β1 integrin cytoplasmic tail, including the stop codon from exon 16 (called Cyto-cDNA), in frame into exon 15 (see Figure S1A in Supporting Information). In addition, a floxed neo-tk cassette was introduced into the targeting vector (*hpm*KI^neo-tk+^). The *hpm*KI^neo-tk+^ mutation was transferred into embryonic stem (ES) cells by homologous recombination (see Figure S1B, left panel, in Supporting Information), and the neo-tk selection cassette was subsequently removed by transient expression of *Cre* recombinase leaving one loxP site in the non coding region (*hpm*KI^lox^). The *hpm*KI^lox^/+ ES cells were used to generate germline chimeric mice (see Figure S1B, right panel, in Supporting Information). Successful germline transmission was confirmed by genomic PCR (see Figure S1C in Supporting Information) and by sequence analysis (not shown).

**Figure 1:**
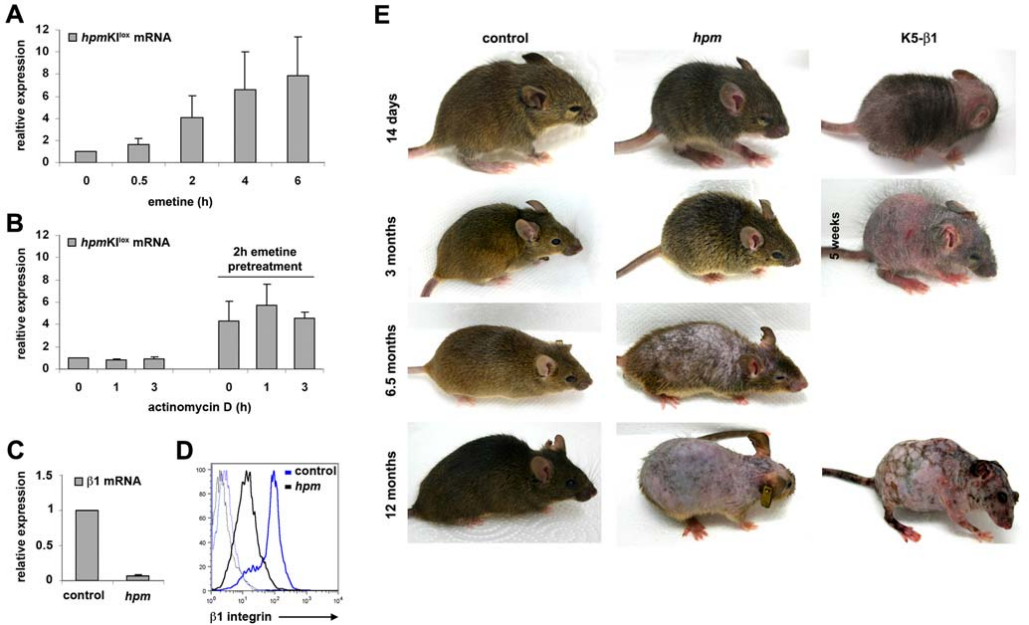
Nonsense-mediated decay (NMD) leads to reduced β1 integrin expression from the *hpm*KI^lox^ allele. (A) Subconfluent primary keratinocytes isolated from adult *hpm*KI^lox^/+ mice were treated with 100 µg/ml emetine to block protein translation and thus the NMD pathway, or (B) treated with 5 µg/ml actinomycin D to block *de novo* mRNA transcription in the presence or absence of emetine. Cells were lysed at indicated times, RNA isolated and applied as template for quantitative RT-PCR using primers specific for the *hpm*KI^lox^ cDNA. Quantification of 3 independent experiments is shown. Error bars indicate standard deviations (s.d.). (C) Quantification of RT-PCR analysis using primers specific for total β1 mRNA shows reduced transcript levels in 3-week-old *hpm* epidermis when normalized to controls. RNA from 5 mice per genotype was used as template for the analysis. Error bars indicate s.d. (D) Representative histogram of flow cytometry analysis reveals that cell surface expression of β1 integrin is reduced on freshly isolated keratinocytes from 3-week-old *hpm* mice. Blue and black histograms denote control and *hpm* keratinocytes, respectively, and background fluorescence is shown as dotted line. The flow cytometry experiments were performed with 3 mice of each genotype. (E) *hpm* mice show progressive hair loss, which is delayed and less severe when compared with K5-β1 mice. The age of the mice is indicated.

The stop codon in exon 15 of the *hpm*KI^lox^ allele should give rise to a transcript with a premature translation termination signal 200 nucleotides upstream of the exon-junction site generated upon splicing of exons 15 with exon 16. Consequently, this additional stop codon located 5′ relative to the last exon of the β1 integrin gene should significantly downregulate the *hpm*KI^lox^ mRNA by the nonsense mediated decay (NMD) pathway [15]. To test whether the *hpm*KI^lox^ mRNA was indeed subjected to NMD, we first analyzed the level of *hpm*KI^lox^ mRNA in primary *hpm*KI^lox^/+ keratinocytes treated with the translation inhibitor emetine to abrogate the NMD pathway [16], [17]. As expected, incubation of subconfluent *hpm*KI^lox^/+ keratinocytes with emetine revealed that the *hpm*KI^lox^ mRNA levels steadily increased with time of treatment suggesting that the *hpm*KI^lox^ mRNA is short-lived and can be stabilized by emetine (Figure 1A). To test whether the emetine-mediated β1 mRNA up-regulation is caused by inhibition of NMD or by an emetine triggered stress response [18], [19] primary *hpm*KI^lox^/+ keratinocytes were treated with the transcriptional inhibitor actinomycin D either in the presence or absence of emetine. Actinomycin D treatment influenced neither the levels of non-stabilized *hpm*KI^lox^ mRNA nor the levels of emetine-stabilized *hpm*KI^lox^ mRNA (Figure 1B) indicating that the increase of emetine-triggered *hpm*KI^lox^ mRNA is caused by the inhibition of the NMD pathway.

### Reduction of β1 integrin levels on keratinocytes results in an attenuated β1 integrin null-like phenotype

Mice heterozygous for the *hpm*KI^lox^ mutation (*hpm*KI^lox^/+) were normal. Heterozygous intercrosses revealed that homozygous (*hpm*KI^lox^/*hpm*KI^lox^) mice die at around E6-6.5 (embryonic day 6–6.5) (Table 1), while mice with a complete ablation of the β1 integrin gene showed an empty implantation chamber at this developmental stage, suggesting that homozygous *hpm*KI^lox^/*hpm*KI^lox^ mice survive slightly longer [20], [21]. These data indicate that reduced β1 integrin levels allow the embryonic development to proceed further than in the complete absence of β1 integrin expression.

**Table 1:** Genotypes of progeny from heterozygous intercrosses.

		*hpm*KI^lox^/*hpm*KI^lox^		*hpm*KI^lox^/+		+/+	
Stage	Total	n	%	n	%	n	%
E6.5	55	13^a^	23.6	31	56.4	11	20

a all embryos were extensively degenerated at this developmental stage

To test whether the reduced expression of β1 mRNA from the *hpm*KI^lox^ allele is affecting skin homeostasis, we generated mice solely expressing the *hpm*KI^lox^ allele in keratinocytes. To this end, we intercrossed the *hpm*KI^lox^/+ and floxed β1 integrin strains with transgenic mice expressing the Cre recombinase under the Keratin 5 (K5) promoter [6], [22] to generate *hpm*KI^lox^/β1fl; K5Cre (*hpm*) mice. The *hpm* mice lose the β1 floxed allele in Cre expressing cells and thus, express only the *hpm*KI^lox^ allele in keratinocytes of the IFE and the outer root sheath (ORS) of hair follicles (HFs). To determine the expression of integrins in 3-week-old *hpm* keratinocytes we measured β1 mRNA levels with quantitative Real-Time (RT) PCR and integrin surface levels with flow cytometry. The β1 mRNA levels were reduced by 93±2% in the *hpm* mice (Figure 1C), and the cell surface levels of β1 and α2 integrins were reduced by 85.9±3% and 65.1±19%, respectively, compared to control cells (Figure 1D and see Figure S2 in Supporting Information). The expression of the hemidesmosomal integrins α6 and β4 was slightly increased (see Figure S2 in Supporting Information). These data demonstrate that subjecting β1 integrin mRNA to NMD leads to severely reduced β1 integrin levels.

Next we investigated whether ∼15% of the normal β1 integrin levels are sufficient to prevent the development of skin abnormalities, which were observed in mice lacking the β1 integrin gene in keratinocytes (K5- β1)[6]. In agreement with our previous report, K5-β1 mice had a reduced number of hairs at 2 weeks of age and almost no hair at the age of 4–5 weeks. In contrast, *hpm* mice had only a slightly thinner fur at the age of 2 weeks and lost their hair coat between 6 and 12 months of age (Figure 1E, and data not shown). Also in sharp contrast to K5-β1 mice, *hpm* mice never developed wounds, moved unrestrained and were fertile.

**Figure 2:**
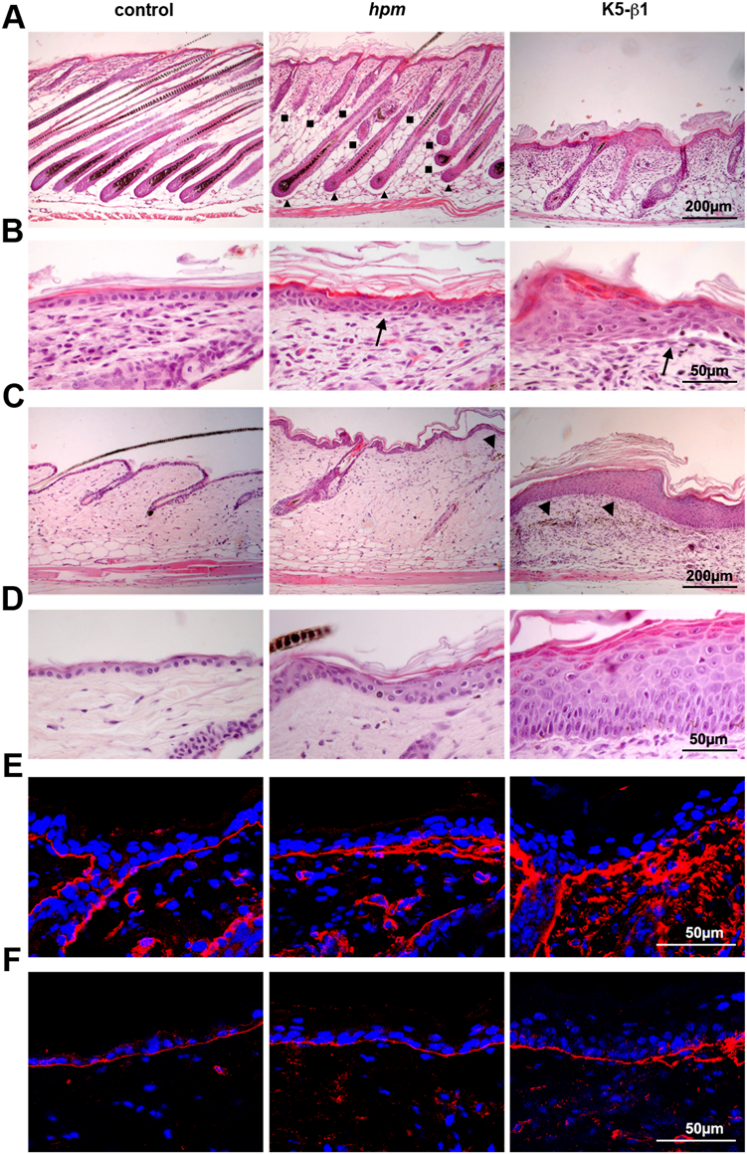
Epidermal and HF phenotype in *hpm* mice. (A, B) Haematoxylin-eosin stained sections from the back skin of 14-day-old control, *hpm* and K5-β1 mice. (A) *hpm* mice displayed stunted HF morphogenesis leading to two HF types (▴ fully developed with multilayered ORS; ▪ shortened, malformed and prematurely arrested in morphogenesis), while in K5-β1 mice all HFs were severely distorted. (B) Control epidermis consists of a monolayer of cuboidal cells firmly attached to the dermis, while *hpm* epidermis consisted of 2–3 keratinocyte layers with few microblisters at the BM zone (arrows). The K5-β1 epidermis consisted of 2–7 layers of roundish, polygonal or flattened keratinocytes that were frequently detached from the dermis forming large blisters (arrows). (C, D) Haematoxylin-eosin stained sections from the back skin of 6.5-month-old control and *hpm* and of 5-week-old K5-β1 mice. (C) Progressive hair loss in *hpm* epidermis is accompanied by the development of dermal fibrosis with scattered melanin deposits (arrowheads). The remaining HFs are severely malformed. In K5-β1 mice the dermal fibrosis developed already at 5 weeks of age and was accompanied by an almost complete loss of HFs and large melanin deposition in the dermis (arrowheads). (D) The epidermis of the back skin of 6.5-month-old *hpm* mice is hyperthickened but has almost no microblisters. The epidermis of 5-week-old K5-β1 mice is severely hyperthickened and shows reduced blistering when compared to 2-week-old skin. (E, F) Immunostaining for LM-332 of back skin sections. (E) At 14 days of age LM-332 (red) is diffusely distributed at the dermal-epidermal junction of the *hpm* mouse skin, however less pronounced than in K5-β1 epidermis. Nuclei were stained with DAPI (blue). (F) At 6.5 months of age LM-332 (red) shows a largely normal, linear deposition at the dermal-epidermal junction of *hpm* skin. LM-332 deposition is also improved in 5-week-old K5-β1 epidermis. Nuclei were stained with DAPI (blue).

Histology revealed that 14-day-old *hpm* back skin had a normal number of HFs. However, the morphology of *hpm* HFs was abnormal. About 60% of the *hpm* HFs were misshapen and arrested in morphogenesis resembling the K5-β1 phenotype, while about 40% reached down to the muscle layer and displayed a severely abnormal and multilayered ORS (Figure 2A). The *hpm* IFE contained 2–3 layers of nucleated cells (epidermal hyperplasia), occasional small microblisters at the dermal-epidermal junction (Figure 2B, arrows) and laminin-332 (LM-332) deposits reaching into the dermis (Figure 2E). Similar, but more severe defects were observed in K5-β1 skin (Figure 2A,B,E).

**Figure 3:**
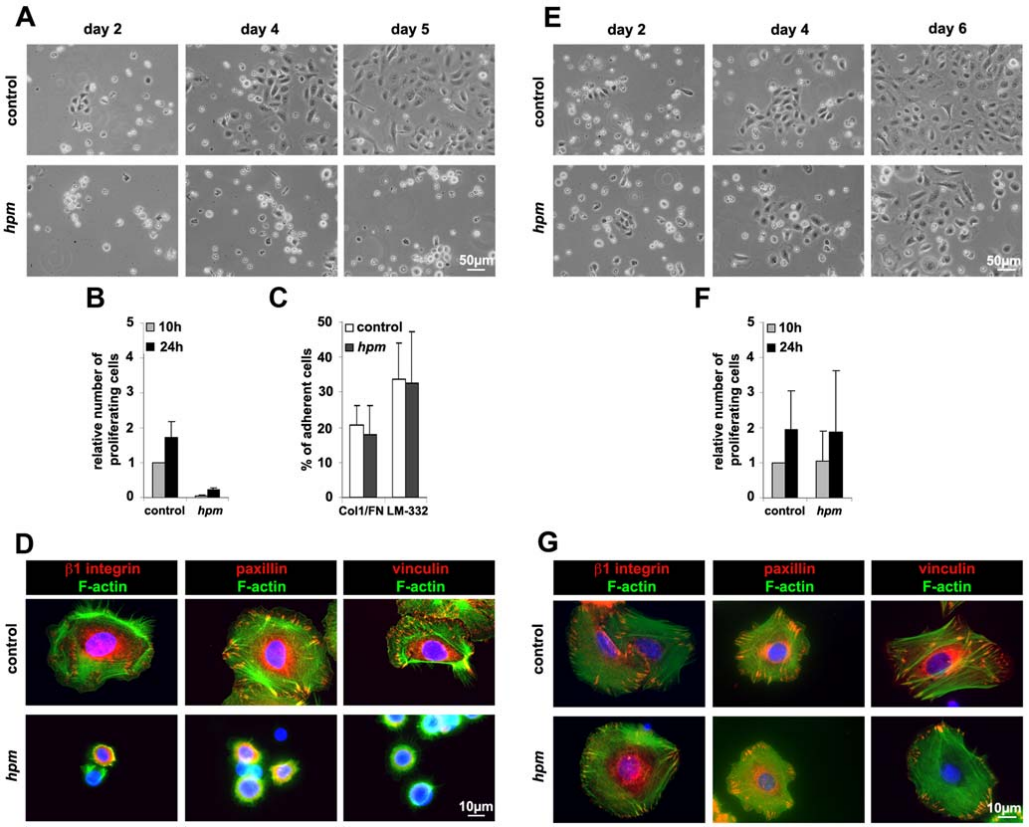
Reduced spreading defect of keratinocytes from old *hpm* mice. (A) Freshly isolated keratinocytes from 2.5-month-old mice seeded on a mixture of Col1 and FN. In contrast to control cells, *hpm* cells neither spread nor grow *ex vivo*. Time points after seeding are indicated. (B) Proliferation rate of keratinocytes from 3-week-old mice cultured for 2 days on Col1/FN and pulse-labelled with BrdU for 10 or 24 hours. Compared to control cells *hpm* keratinocytes have significantly reduced proliferation rate. Bars represent data from keratinocytes of 3 mice per genotype. Error bars indicate s.d. (C) Cell adhesion of *hpm* keratinocytes from 2.5-month-old mice plated on Col1/FN or on LM-332 is similar to control cells. 5 and 4 independent experiments were performed on Col1/FN or LM-332, respectively. Error bars indicate s.d. (D) Keratinocytes from 2.5-month-old control and *hpm* mice were cultured for 2 days on Col1/FN and immunostained for β1 integrin, paxillin and vinculin (red), and F-actin (phalloidin; green). Nuclei were stained with DAPI (blue). *hpm* cells failed to develop focal adhesions and to organize the actin cytoskeleton into stress fibers. (E) Freshly isolated keratinocytes from 7.5-month-old mice plated on Col1/FN. Both control and *hpm* keratinocytes were well spread and showed a similar proliferation rate. (F) Control and mutant keratinocytes isolated from 6.5-month-old mice plated on Col1/FN and cultured for 2 days were pulse-labelled with BrdU and harvested at times indicated. No significant difference in BrdU incorporation was observed between control and *hpm* keratinocytes. Bars represent data from at least 3 mice per genotype. Error bars indicate s.d. (G) Keratinocytes isolated from 7.5-month-old control and *hpm* mice were cultured for 2 days on Col1/FN and immunostained for β1 integrin, paxillin and vinculin (red) and F-actin (phalloidin; green). Nuclei were stained with DAPI (blue). Size and number of FAs and actin stress fibers is similar in *hpm* and control keratinocytes.

To assess whether the reduced β1 integrin levels on *hpm* keratinocytes are sufficient for in vitro functions we isolated keratinocytes from 2.5-month-old wt and *hpm* mice and seeded them on a surface coated with a mixture of collagen I (Col1) and fibronectin [23]. While the majority of adherent wt cells began spreading one day after plating and grew to confluency 4 days later, *hpm* cells adhered but failed to spread and proliferate (Figure 3A). To determine the proliferation rates of wt and *hpm* keratinocytes we performed an ELISA-based BrdU incorporation assay and could not find proliferating *hpm* cells on Col1/FN surfaces (Figure 3B). To quantify adhesion, we plated wt and *hpm* keratinocytes on a mixture of Col1/FN or LM-332, respectively, and observed similar adhesion efficiencies in both genotypes (Figure 3C). Next we compared focal contact organization and F-actin distribution between wt and *hpm* cells after a culture period of 2 days on a Col1/FN mixture. Immunostaining for β1 integrin, vinculin and paxillin, and visualization of F-actin with fluorescently labeled phalloidin revealed a complete absence of FA structures and a failure to organize F-actin into stress fibres in *hpm* keratinocytes (Figure 3D). The immunosignals of FA proteins were observed in the cytoplasm and F-actin appeared as clumps or fine filaments extending from the perinuclear region. Altogether these findings indicate that ∼15% of the normal β1 integrin level is not sufficient to support normal epidermal and HF homeostasis in vivo and integrin functions in vitro.

### The defects of epidermal *hpm* keratinocytes ameliorate with age

In agreement with the external appearance (Figure 1E), histology of skin sections from 6.5 months old *hpm* mice revealed reduced and misshapen HFs, a mild dermal fibrosis, and dermal melanin deposits from perished HFs (Figure 2C). Surprisingly, we observed that while the hyperplasia of the IFE remained similar, the epidermal-dermal blisters almost completely disappeared (Figure 2D). Similarly, the LM-332 deposition appeared either normal or extended to a much lesser extent into the dermis (Figure 2F). Interestingly, the blistering and BM abnormalities ameliorated also in the skin of older K5-β1 mice (Figure 2C,D,F).

When we performed adhesion and spreading assays with freshly isolated keratinocytes isolated from 6.5–7.5 months old animals, we observed that both wt and *hpm* keratinocytes were adhering, spreading and proliferating with an apparently comparable kinetics (Figure 3E). The similar proliferation rates were confirmed with BrdU incorporation assays (Figure 3F). Furthermore, the *hpm* keratinocytes formed normal FAs containing β1 integrins, vinculin and paxillin and had a normal F-actin network (Figure 3G).

These findings indicate that the epidermal defects caused by the *hpm* allele ameliorate with age.

### β1 integrin levels increase on *hpm* keratinocytes with age and upon skin wounding

**Figure 4:**
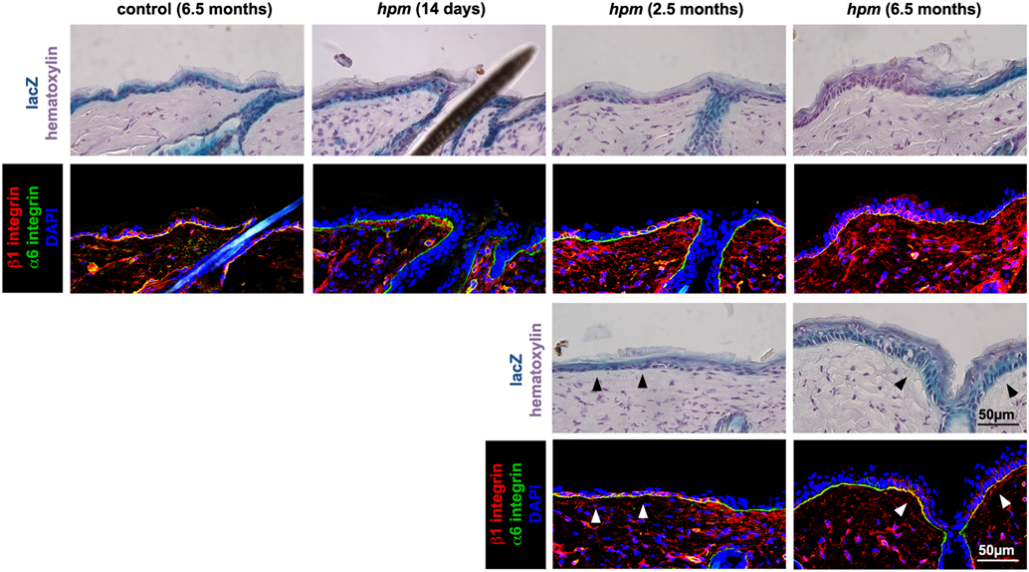
Increased number of β1 integrin expressing epidermal keratinocytes in skin of old *hpm* mice. Consecutive sections of back skin from 14-day-, 2.5-month- and 6.5-month-old *hpm* mice and 6.5-month-old control mice were immunostained for β1 (red) and α6 integrin (green) and examined for lacZ expression as readout for Cre-mediated β1 integrin gene deletion. Sections showing lacZ signals were counterstained with haematoxylin to visualize skin morphology. Staining of back skin of 14-day-old *hpm* mice revealed that the majority of cells in the epidermis and HFs is positive for lacZ and negative for β1 integrin expression. Back skin from 2.5- and 6.5-month-old *hpm* mice revealed regions with lacZ-negative and β1 integrin-positive keratinocytes and regions with lacZ-positive and β1 integrin-positive keratinocytes in the interfollicular epidermis (arrowheads), while in control skin the entire epidermis was lacZ-positive and β1 integrin-positive.

We hypothesized that the amelioration of in vivo and in vitro defects in keratinocytes from aging *hpm* and K5-β1 mice, respectively, could be due to the expansion of keratinocytes that escaped NMD or Cre-mediated deletion of the floxed allele and had elevated β1 integrin levels. To test whether *hpm* keratinocytes can indeed increase their integrin levels we immunostained sections from the back skin of *hpm* mice. The immunostaining revealed that even though β1 integrin signals were not detectable in 14-day-old *hpm* skin, small cell nests with strong β1 integrin expression became apparent in the basal keratinocyte layers of 2.5-month-old *hpm* skin (Figure 4). Interestingly, the number of cells expressing high levels of β1 integrin increased further in 6.5 months old *hpm* epidermis (Figure 4).

To determine whether the rise of β1 integrin expressing cells was due to an incomplete loss of β1 floxed alleles, we used adjacent skin sections from both wt and *hpm* mice to compare β1 integrin and *lacZ* expression, which is activated upon deletion of the β1 integrin floxed allele in control and *hpm* mice [6]. Sections from control skin revealed that irrespective of the age all keratinocytes expressed β1 integrins and were positive for lacZ (Figure 4 and not shown). The epidermis from 14 days old *hpm* mice was lacZ-positive and lacked visible β1 integrin expression (Figure 4). In contrast, the epidermis of 2.5- and 6.5-months-old *hpm* mice contained lacZ-negative regions of variable size (Figure 4). Several lacZ-negative regions were β1 integrin-positive indicating that the epidermis contains stretches of keratinocytes, which have escaped Cre-mediated deletion of the floxed β1 integrin allele (Figure 4). However, we also observed keratinocytes in the *hpm* epidermis that were both, lacZ- and β1 integrin-positive, suggesting that they failed to downregulate the *hpm*KI^lox^ mRNA by the NMD pathway (Figure 4, see arrowheads).

**Figure 5:**
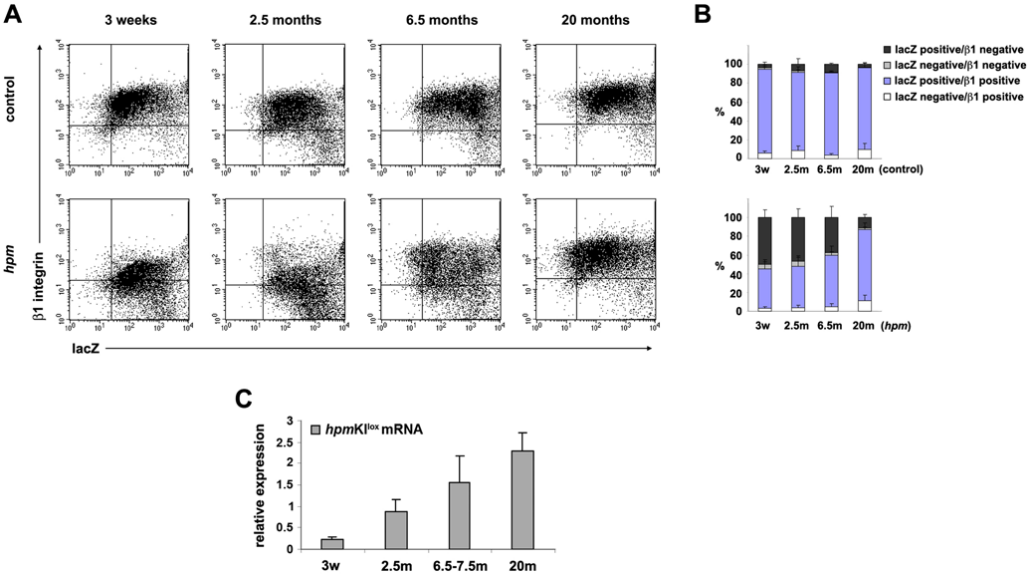
Keratinocytes escaping NMD increase β1 integrin expression and improve skin defects in old *hpm* mice. (A) Expression of lacZ and β1 integrin in primary keratinocytes from 3-week-, 2.5-month-, 6.5-month- and 20-month-old control and *hpm* mice assessed by flow cytometry. Keratinocytes were stained with an anti-β1 integrin antibody and subsequently incubated with a fluorogenic substrate (fluorescein di-β-D-galactopyranoside) to determine β-galactosidase activity. The expression of β1 integrin increases with the age of *hpm* mice. (B) Quantification of results from flow cytometry from at least 3 mice per genotype and developmental stage. Error bars indicate s.d. (C) Expression of the *hpm*KI^lox^ transcript determined by quantitative RT-PCR analysis. RNA isolated from epidermal lysates from control and *hpm* mice at indicated ages was used as template for quantitative RT-PCR analysis with primers specific for the *hpm*KI^lox^ RNA. Expression of the *hpm*KI^lox^ RNA in *hpm* mice is shown relative to the expression levels of the *hpm*KI^lox^ RNA in control mice. The abundance of the *hpm*KI^lox^ transcript in *hpm* mice increases with age relative to control mice. 1 to 4 control and 3 to 5 *hpm* mice per age were analysed. Error bars indicate s.d.

To confirm these findings we determined the expression level of the β1 integrin subunit and lacZ by flow cytometry in keratinocytes from 3-week-, 2.5-month-, 6.5-month- and 20-month-old control and *hpm* mice. As shown in Figure 5A and B, β1 integrin expression gradually increased to normal levels in *hpm* keratinocytes from 3-week-old to 20-months-old mice. Interestingly, the majority of β1 integrin-positive *hpm* keratinocytes expressed high levels of lacZ corroborating that NMD of *hpm*KI^lox^ mRNA was reverted in ageing *hpm* mice. To further confirm this finding we isolated RNA from epidermal lysates from 3-week-, 2.5-months-, 6.5-7.5-months- and 20-months-old control and *hpm* mice and compared their *hpm*KI^lox^ mRNA levels using quantitative RT-PCR. We observed a significant increase in *hpm*KI^lox^ mRNA between 2.5- and 20-month-old *hpm* mice relative to littermate controls (Figure 5C). Finally, the increase in *hpm*KI^lox^ mRNA concomitantly led to an increase in cell surface levels of β1 integrin-associated α subunits on *hpm* keratinocytes (see Figure S2 in Supporting Information). Flow cytometry of primary keratinocytes from 3-week-, 2.5-months-, 6.5-months- and 20-months-old *hpm* mice revealed that the surface expression of α2 integrin steadily increased and reached control levels in cells from 20-months-old mice (see Figure S2 in Supporting Information, upper panel). The expression of α6 and β4 integrins slightly exceeded that seen in control animals, both in young and old *hpm* mice (see Figure S2 in Supporting Information, lower two panels).

Finally, we tested whether the expansion of β1^high^ cells is associated with an increased number of proliferating keratinocytes. At 14 days of age the percentage of BrdU-positive cells was similar in control and *hpm* mice (10±3.2% in *hpm* and 10±1.9% in control epidermis), while at 6.5 months of age the percentage of BrdU positive cells was significantly higher in *hpm* skin (3.9±0.7%) than in control skin (1.5±0.7%). Altogether these findings indicate that keratinocytes escaping NMD expand in the epidermis of aged *hpm* mice due to their increased proliferative potential.

We next determined if these escape mechanisms are also active in healing skin wounds and if the wound-induced hyperproliferation accelerates this process. To test this possibility we generated full-thickness excision wounds in *hpm* mice and wild-type littermates at the age of 8–12 weeks. Surprisingly, the rate of wound closure as well as the area of hyperproliferative epidermis was not reduced in *hpm* mice at day 5 after injury (see Figure S3A in Supporting Information). At day 13 after wounding, wounds in mice of both genotypes were fully reepithelialized. The only difference that we observed was an enlarged width of the healed wound and accordingly, an increased area of wound epidermis (see Figure S3B in Supporting Information). This phenotype most likely results from reduced contraction of the wound due to fibrosis in the dermis. However, the rate of reepithelialization was not affected. When sections from 13-day wounds were stained for β-galactosidase activity, large areas of the wound epidermis above the healing wound were found to be lacZ-negative (see Figure S3C in Supporting Information), demonstrating that these cells escaped Cre-mediated recombination. This finding suggests that the necessity for rapid keratinocyte proliferation upon skin injury accelerates the selection process that we also observed in ageing skin.

### β1 integrin-positive keratinocytes increase in K5-β1 epidermis with age

**Figure 6:**
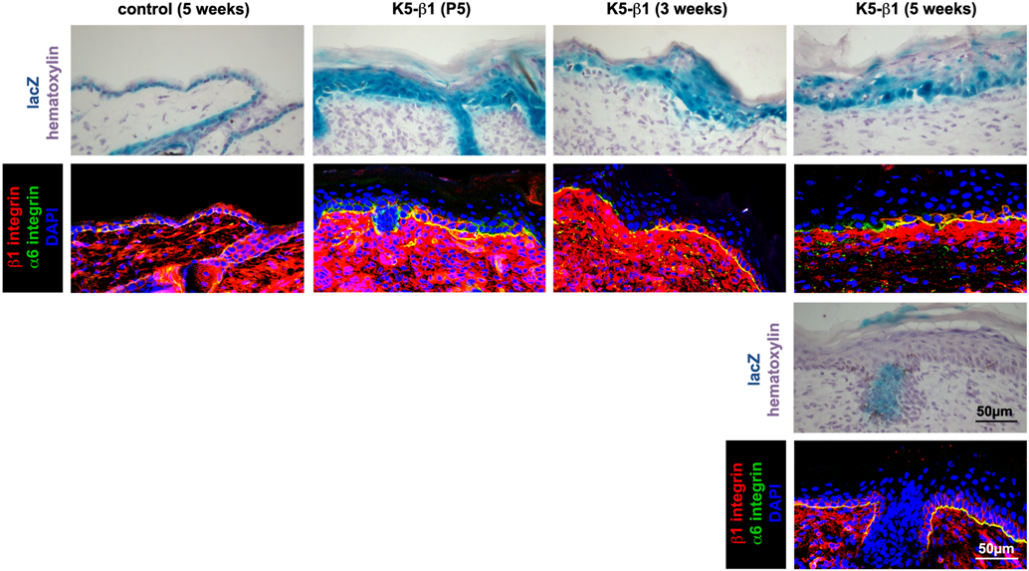
Escape from Cre-mediated recombination leads to partial rescue of defects in K5-β1 skin. Consecutive sections from the back skin of 5-day-, 3-week- and 5-week-old K5-β1 and 5-week-old control mice were immunostained for β1 integrin (red) and α6 integrin (green), and examined for Cre-induced lacZ expression. Sections were counterstained with haematoxylin. Staining of back skin from 5-day-old (P5) K5-β1 mice revealed that the majority of cells in the epidermis and HFs is positive for lacZ and negative for β1 integrin expression. The IFE rarely contains a few β1 integrin-positive and lacZ-positive cells. At 3 weeks of age the areas with lacZ-positive and β1 integrin-positive cells increased. At 5 weeks of age the majority of the IFE was β1 integrin-positive with some cells showing lacZ activity (one β1 floxed allele deleted) and some showing no detectable lacZ activity (both β1 floxed alleles escaped Cre-mediated recombination).

To test whether the partial rescue of the skin phenotype in 5-week-old K5-β1 mice was caused by the expansion of β1 integrin-positive cells, we analyzed adjacent back skin sections from 5-day-, 3-week- and 5-week-old control and K5-β1 mice for integrin and lacZ expression. At the age of 5 days the K5-β1 back skin was positive for lacZ and only single cells or very small cell nests in the IFE also expressed β1 integrin (Figure 6, and data not shown). In 3-week-old K5-β1 mice an increasing number of β1 integrin-positive keratinocytes was observed in lacZ-positive areas. By 5 weeks of age large regions of the K5-β1 IFE were β1 integrin positive. Interestingly, these areas were either lacZ-positive (Figure 6, upper panel) or negative (Figure 6, lower panel) in the adjacent sections. These results suggest that the β1 integrin-positive cells can result from inefficient recombination of either one (β1 integrin-positive and lacZ-positive) or both (β1 integrin-positive and lacZ-negative) β1 floxed alleles.

**Figure 7:**
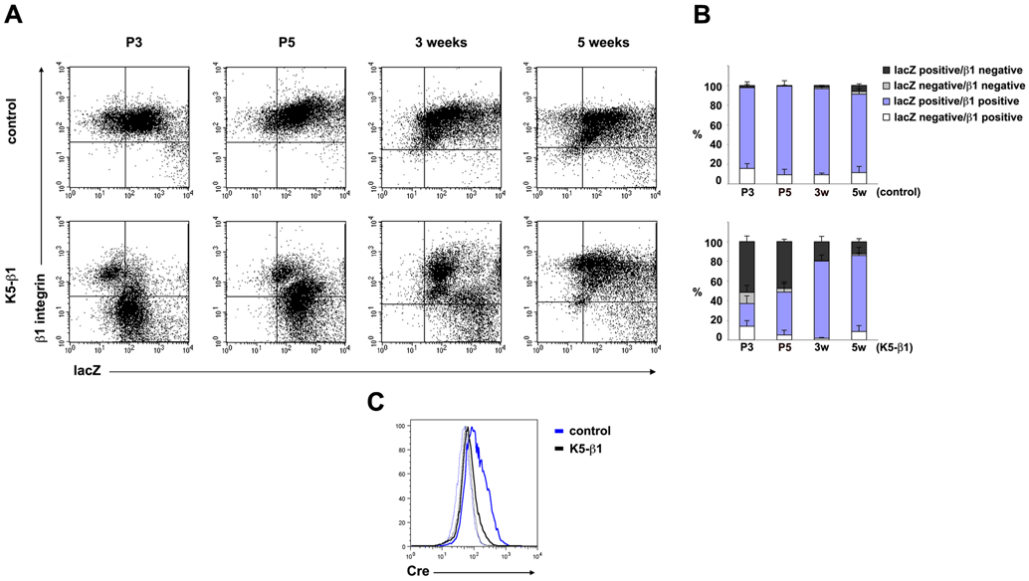
Keratinocytes with impaired deletion of β1 gene expand and partially rescue defects of K5- β1 mice. (A) Cell surface expression of β1 integrin and lacZ activity in freshly isolated keratinocytes from 3-day (P3) 5-day- (P5), 3-week- and 5-week-old control and K5-β1 mice assessed by flow cytometry. Keratinocytes were stained with an anti-β1 integrin antibody and subsequently assessed for β-galactosidase activity. The number of β1 integrin expressing cells is increasing in aging K5-β1 mice. (B) Quantification of results from flow cytometry from at least 3 mice per genotype and developmental stage. Error bars indicate s.d. (C) Expression of the Cre transgene in β1 integrin expressing cells determined by flow cytometry. Primary keratinocytes from 5-week-old control (β1^fl/+^/K5Cre) and K5-β1 mice were stained with an anti-β1 integrin and an anti-Cre antibody. Cre expression in cells gated for high β1 integrin expression is shown in the histogram. Cre expression was dramatically reduced in K5-β1 keratinocytes compared to control keratinocytes. The results represent the analysis of 3 age-matched control and K5-β1 mice. Blue and black histograms denote control and K5-β1 keratinocytes, respectively. The isotype controls are shown as dotted lines.

To confirm and quantify the immunohistochemical analysis we performed flow cytometry on freshly isolated keratinocytes from 3-day, 5-day-, 3-week- and 5-week-old control and K5-β1 mice. We could clearly show that the β1 integrin expressing cell population increased with age in K5-β1 mice from 37.3%±2.6 at 3 days after birth to 85.8%±3.7 at 5 weeks of age (Figure 7A,B). Remarkably, the vast majority of the β1 integrin-positive cells was lacZ-positive suggesting that in most cells only one β1 floxed allele was deleted.

To define why there was inefficient β1 integrin gene deletion, we measured β1 integrin and Cre levels in primary keratinocytes from 5-week-old control (β1^fl/+^/K5Cre) and K5-β1 mice. We found that high β1 integrin expression levels correlated with low Cre levels in the K5-β1 mice (Figure 7C) suggesting that low Cre transgene expression leads to inefficient recombination of the floxed β1 integrin allele, perpetuation of normal β1 integrin expression on basal keratinocytes, their expansion and finally amelioration of the defects.

## Discussion

β1 integrins are essential for tissue development and maintenance [1], [20], [24]. They are highly expressed on stem and progenitor cell populations, which orchestrate organogenesis and represent a cellular reservoir to maintain organ homeostasis [25], [26]. Experimental evidence suggests that the high expression level is critically important for supporting stem and progenitor cell properties such as adhesion to their niche, cell cycle quiescence and the appropriate orientation of the mitotic spindle to control asymmetric versus symmetric cell division [27]–[30]. Although the function of β1 integrins for morphogenesis is undisputed, their role in stem and progenitor cell maintenance and expansion is less clear. Cell type-specific deletions of the β1 integrin gene in the skin, mammary gland and haematopoietic system revealed a differential requirement of the β1 integrin subfamily for stem and progenitor function [6], [7], [30]–[33]. While deletion of the β1 integrin gene had no apparent impact on resting hematopoietic stem and progenitor cells, deletion of the β1 integrin gene in keratinocytes reduced the proliferation rate of skin progenitor cells and in the mammary gland epithelium severely affected both, the proliferation and asymmetric division of the stem and progenitor cell population [30].

In the present paper we generated a mouse strain expressing reduced β1 integrin levels to directly investigate the role of β1 integrin on skin formation and homeostasis, avoiding gross damages as seen in complete knockout animals. Since the β integrin subunits are usually expressed in excess, the expression of α/β integrin heterodimers on the cell surface is controlled by the available amount of α integrin subunits. This is the reason why mice heterozygous for the deletion of the β1 integrin gene have normal or only slightly reduced levels of αxβ1 integrins on their cells and are thus phenotypically normal. To significantly reduce the expression level of β1 integrins we decided to impair the stability of the β1 integrin mRNA. To this end we fused the coding sequence of the last exon to the second-last exon of the β1 integrin gene. We thereby introduced a premature translation termination codon into the second-last exon, which in turn induced the NMD pathway and reduced β1 integrin levels on keratinocytes to ∼15%. Mice homozygous for the hypomorphic β1 integrin allele die between E6.0–E6.5, indicating that the remaining integrins allow development to proceed slightly further than the complete β1 gene ablation, as such mice die at the late blastocyst stage [20]. We are currently trying to identify the specific defect(s) underlying the lethality of mice homozygous for the *hpm*KI^lox^ allele.

Skin development and maintenance depend on the presence of epithelial progenitor or stem cells, which retain their proliferative capacity throughout the entire life span of an animal. They have to replenish the interfollicular epidermis and the hair follicles and are therefore crucial to maintain the skin and the hairs. Genetic studies in mice have shown that the ablation of the β1 integrin gene (K5-β1 mice) or the expression of inactive β1 integrins on keratinocytes leads to severe skin and hair coat phenotypes [6], [7], [34]. The defects include progressive hair loss, malformation of the hair follicles, hyperthickened and detached epidermis, defective LM-332 deposition at the dermal-epidermal junction, reduced keratinocyte proliferation and dermal fibrosis. The *hpm* mice with 15% of normal β1 integrin levels develop similar phenotypes, albeit much less severe and delayed. Their IFE epidermis is less thick, blisters are very small and scarce and almost all HF are malformed. Primary keratinocytes isolated from young *hpm* mice adhered well to ECM substrates but failed to spread, to proliferate and to survive in vitro, suggesting that the reduced β1 integrin levels are sufficient to promote adhesion but insufficient to appropriately activate signalling pathway(s) required for F-actin reorganisation, cell spreading and proliferation.

Despite the skin abnormalities *hpm* mice had an apparently normal life span. Old *hpm* mice continued to lose their hair, but microblisters and irregular LM-332 deposition were significantly ameliorated with age. Moreover, primary keratinocytes from old *hpm* were able to spread and organize actin into stress fibers, to form robust and normal numbers of focal adhesions and to proliferate. The reason for the ‘rescue’ of the mutant phenotype in vivo and in vitro was the very rapid expansion of β1 integrin expressing keratinocytes in *hpm* skin. Around 11% of the β1 integrin expressing cells escaped Cre-mediated deletion. Similar findings were obtained in our previous study [6]. Furthermore, ∼76% of β1 integrin expressing keratinocytes escaped or compensated for NMD-mediated degradation of β1 integrin mRNA. These results indicate that keratinocytes with high β1 integrin levels have an enormous advantage over cells expressing low β1 integrin levels, leading to their rapid expansion in the mutant skin and partial reversion of the epidermal phenotype. Interestingly, this advantage becomes even more pronounced in situations where the requirement for keratinocyte proliferation and migration is particularly high such as during wound healing.

Concomitant with the restoration of the β1 integrin expression in the mutant animals there was also an increase in cell surface expression of its dimerization partner α2 integrin that was strongly reduced in young animals. On contrary the expression of the hemidesmosomal integrins α6 and β4 remained relatively stable throughout the entire life span of the *hpm* mice slightly exceeding that of aged-matched control littermates. These data suggest that the α6β4 integrins do not play a major role in the regulation of the proliferative potential of the keratinocytes, which only increases once cells expressing normal levels of β1 integrin repopulate the skin.

The K5-β1 mice carry disrupted β1 integrin genes in keratinocytes, resulting in loss of β1 integrin expression shortly after birth in around 60–70% of basal keratinocytes. Most K5-β1 mice die within the first three weeks after birth. A few mice survive beyond the age of 5 weeks, despite their severe skin abnormalities. Interestingly, the epidermal phenotype of the 5 weeks old K5-β1 mutant also improved indicated by a reduced number of blisters at the epidermal-dermal junction and normal deposition of LM-332. This phenotype improvement is also due to expansion of β1 integrin expressing cells; already 5 days after birth the number of β1 integrin exprerssing cells increased to ∼50%, and at the age of 5 weeks to ∼86%. The majority of these cells were still lacZ-positive indicating that in the majority of β1 integrin-positive cells only one β1 integrin floxed allele was excised by the K5-Cre transgene. These data indicate that the proliferative potential of β1 integrin expressing keratinocytes is so superior that they overgrow β1 integrin-null keratinocytes in a few weeks leading to a partial rescue of the interfollicular phenotype.

In contrast to the partial rescue of the interfollicular phenotype, the hair follicle phenotype was progressive in the mutant animals, leading to a complete hair loss in 1 year old *hpm* and 5-week-old K5-β1 mice. This finding suggests that once a hair follicle is malformed and has lost the connection to the dermal papilla, it is no longer able to fully regenerate independently of the presence of β1 integrin re-expressing EPCs in the skin.

Our findings fully support previous in vitro data showing that high expression of β1 integrins on keratinocytes is important for stem or progenitor cell-like properties [9]–[12]. Their role for stem/progenitor cells was questioned when mice with a deletion of the β1 integrin gene in keratinocytes maintained an epidermis without premature terminal differentiation [6]. The analysis of the β1 hypomorph mice and of surviving β1 integrin deficient mice revealed that β1 integrins are enormously important for the self-renewal capacity of the proliferating cells of the epidermis [35]. The K5-β1 mice surviving beyond 5 weeks [6] benefit from the enormous proliferative potential conferred by β1 integrin-mediated adhesion and signals. Interestingly, ablation of the floxed β1 integrin allele differs in other cell systems such as the hematopoietic organ. Deletion of the β1 integrins has no severe impact on hemtaopoietic stem cell/progenitor cell proliferation, lineage commitment and lineage expansion. It will require further studies to explain why keratinocytes so critically depend on the function of this integrin subfamily while blood cells apparently do not.

## Materials and Methods

### Ethics Statement

All animal studies were approved by the Regierung von Oberbayern (Germany) or the veterinary authorities of Zurich (Switzerland).

### Generation of mutant mice

Mouse genomic clones used to generate the targeting construct were described earlier [20]. The loxP flanked neomycin resistance - thymidin kinase gene (neo-tk) selection cassette was inserted together with a 141 bp wt cDNA fragment of the β1 integrin gene coding for the entire cytoplasmic tail of the β1 integrin chain including the stop codon, into exon 15, just behind and in frame with the transmembrane coding region. The targeting construct (see Figure S1A in Supporting Information) was electroporated into R1 embryonic stem (ES) cells (129/Sv) and homologous recombinants were identified by Southern blot analysis of *BamHI* -digested DNA with a 5′ external probe (see Figure S1B in Supporting Information ). Positive ES clones were transiently transfected with Cre recombinase to remove the neo-tk cassette, selected in 2′-fluoro-2′-deoxy-1-β-D-arabinofuranosyl-5-iodouracil supplemented growth medium and identified with a 3′ external probe after *BamHI* digestion (see Figure S1B in Supporting Information). Cells that lacked the selection cassette were injected into blastocysts to generate germline chimeras. Male chimeras were intercrossed with C57BL/6 females and offspring carrying the *hpm*KIlox allele were identified by genotyping PCR that distinguishes between the wt and the *hpm*KIlox allele (5′-GTCCTACTGGTCCCGAC-3′, 5′-TGCTCTCAGTAATGTTTCATAAC-3′). The *hpm*KIlox/+ mice were intercrossed with mice carrying a floxed β1 integrin gene [6] and mice carrying a keratin 5 promoter-driven Cre recombinase transgene [22]. For the experiments performed with this mouse line *hpm*KI^lox^/β1^fl^, β1^fl/+^ or β1^fl+^/K5Cre age-matched control animals were used, depending on the requirement of the experiment.

For developmental studies, egg cylinders of heterocygous *hpm*KI^lox^/+ crossings were collected, the embryos isolated and subjected to PCR-based genotyping using the primers described above.

### Isolation of primary keratinocytes, adhesion and proliferation assays

Primary keratinocytes were isolated from mice at indicated ages as described in [36] and either directly applied in a functional assay or seeded on a mixture of 30 µg/ml Vitrogen (bovine collagen type I; Cohesion) and 10 µg/ml fibronectin (Invitrogen)-coated plastic in keratinocyte growth medium containing 8% chelated FCS (Gibco) and 45 µM Ca^2+^.

Cell adhesion of freshly isolated keratinocytes to 5 µg/ml LM-332 (kindly provided by Dr. Monique Aumailley, Cologne University, Germany) or a mixture of 10 µg/ml FN and 30 µg/ml Col1 was assayed as described previously [36], except that 1×10^5^ keratinocytes were seeded per well and that cells were incubated for 2 hours on the depicted ECM substrata, before the assay was developed. Proliferation of primary keratinocytes seeded on a mixture of 10 µg/ml FN and 30 µg/ml Col1 was determined on the second day after plating using a Cell Proliferation ELISA Kit (Roche), according to the manufacturer's protocol. All experiments have been performed in triplicates.

### NMD analysis

The NMD analysis was performed using a modified protocol described by [19]. Subconfluent keratinocytes isolated from adult *hpm*KI^lox^/+ mice were treated with 100 µg/ml of emetine dihydrochloride hydrate (emetine, Fluka) or 5 µg/ml actinomycin D (Sigma Aldrich) for up to 6 and 3 hours, respectively, or pretreated for 2 hours with 100 µg/ml emetine and then incubated for up to 3 hours with 5 µg/ml actinomycin D. Cell pellets were lysed using the TRIZOL Reagent (Invitrogen).

### RNA isolation and Real-Time PCR

Total RNA was extracted from freshly isolated or cultured keratinocytes using TRIZOL Reagent (Invitrogen) and 1 µg of the total RNA was reverse-transcribed using the iScript Synthesis Kit (BioRad), according to the manufacturer's protocol. The single strand cDNA was used as a template for the Real-Time PCR reaction using 2 sets of primers (forward primer hybridizing to exon 14 of the β1 integrin gene (5′- AGGACATTGATGACTGCTGG-3′) and a reverse primer hybridizing to the loxP site and a linker region of the *hpm*KI^lox^ allele (5′-TATGATCGGAATTCCTGCAG-3′) to detect the *hpm*KI^lox^ mRNA and a forward primer hybridizing to the exon 5 - exon 6 border of the β1 integrin gene (5′-AGACTTCCGCATTGGCTTTG-3′) and a reverse primer binding within exon 6 of the β1 integrin gene (5′-GCTGGTGCAGTTTTGTTCAC-3′) to detect total β1 mRNA levels) and the iQ SYBR Green Supermix (BioRad). The reaction was performed in triplicates in an iCycler (BioRad) and evaluated using the software provided by the manufacturer. In all experiments GAPDH was used as reference gene. Results shown in Figures 1A, 1B are normalized for untreated cells, while results shown in Figures 1C, 5C are normalized for the control expression levels of the β1 or *hpm*KI^lox^ mRNA at the respective developmental stage.

### Histology and immunfluorescence

Haematoxylin-eosin and immunofluorescence staining, β-galactosidase activity detection analysis on paraffin embedded or frozen sections, as well as immunofluorescence on fixed cells were performed as described previously [36]. BrdU staining was carried out according to the manufacturer's instructions (Roche Diagnostics). The following antibodies were used: β1 integrin (Chemicon); LM-332 (lamininγ2 chain; kindly provided by Dr. Rupert Timpl); BrdU-POD (Roche Diagnostics); paxillin (Transduction Laboratories); α6 integrin-FITC (BD Pharmingen); vinculin (Sigma Aldrich) and phalloidin-Alexa 488 (Molecular Probes). Fluorescence-conjugated secondary antibodies (goat Anti Rat-Cy3, goat Anti Mouse-Cy3 and donkey Anti Rabbit-Cy3) were purchased from Jackson Immunoresearch. Antibodies were diluted according to the recommendation of the manufacturer.

Images were collected by confocal microscopy (DMIRE2; Leica) using the Leica Confocal Software (version 2.5 Build 1227) with 40× oil objective; or fluorescence microscopy (DMRA2; Leica) using the SimplePCI Software (version 5.1.0.0110) with 63× oil objectives; or bright field microscopy (Axiovert; Zeiss), using the IM50 Software with ×10 or ×40 objectives. All images were collected at RT and processed with Photoshop (Adobe).

### Flow cytometry

Freshly isolated keratinocytes (0.5–1×10^6^ and 2×10^6^ cells per sample for detection of cell surface antigens and for intracellular FACS, respectively) were stained with following antibodies: FITC-labelled anti-β1, anti-α2 and anti-α6 integrin, anti-β4 integrin (all from BD Pharmingen), biotin-labelled anti-Cre recombinase (Covance) or biotin-labelled anti-mouse-IgG1k isotype control (BioLegend) and where required visualized with a FITC-labelled anti-rat-IgG_2a_ secondary antibody or Cy5-labelled streptavidin (both BD Pharmingen). The intracellular detection of Cre recombinase was performed using LEUCOPERM™ reagents (AbD Serotec) according to manufacturer's instructions. Stained cells were subjected to fluorescence-activated cell sorting (FACS)-analysis as described previously [36]. To simultaneously analyze lacZ activity and β1 integrin expression in freshly isolated cells, 2×10^6^ keratinocytes per sample were first stained with a PE-labelled anti-β1 integrin antibody (BD Pharmingen) or a PE-labelled anti-hamster-IgG isotype control (BioLegend) and subsequently incubated with a fluorogenic β-galactosidase substrate fluorescein di-β-D-galactopyranoside (Sigma) as previously described for hematopoietic cells [36]. The staining was performed with cells from the *hpm* mice and age-matched *hmp*KI^lox^/β1^fl^ or β1^fl/+^ and β1^fl+^/K5Cre mice. Cells positive for lacZ activity and β1 integrin were gated according to the staining in keratinocytes from *hmp*KI^lox^/β1^fl^ or β1^fl^/+ mice not expressing the lacZ gene.

### Preparation of wound tissue and histomorphometry

Mice (8–12 weeks old) were anesthetized by intraperitoneal injection of ketamine (75 mg/kg)/xylazine (5 mg/kg). Four full-thickness excisional wounds of 5 mm diameter were generated on the back of mice by excising the skin and the rodent-specific subcutaneous muscle *panniculus carnosus* as described previously [37]. Wounds were left uncovered, harvested at different time points after injury and embedded according to standard procedures.

For morphometrical analysis, 7 µm sections from the middle of the PFA-fixed wounds were stained with haematoxylin-eosin and photographed using a Zeiss Axiophot microscope equipped with a HRc camera (Zeiss, Jena, Germany). The area of the hyperproliferative wound epithelium, the wound width, and the percentage of wound closure were determined using the Openlab 3.1.5 software (Improvision Ltd., Basel, Switzerland). Statistical analysis was performed using the unpaired t-test (given the variances were normally distributed) included in the GraphPad Prism4 software package (GraphPad Software Inc., San Diego, CA).

## Supporting Information

Figure S1 Targeting strategy for the hpm allele. (A) Partial map of the β1A wild-type (wt), the hpm knock-in allele before (hpmKIneo-tk+) and after Cre-mediated deletion of the floxed neo-tk cassette (hpmKIlox). External probes used for Southern blotting after BamHI digest and genotyping PCR primers are indicated. Cyto-cDNA, a wt 141 bp cDNA fragment coding for the complete cytoplasmic domain of the β1A integrin gene including the endogenous stop codon; E15cyto, endogenous sequence of exon 15 encoding for the membrane proximal part of the β1 integrin cytoplasmic tail; filled boxes, exons; D, exon D; neo, neomycin resistance gene; tk, thymidine kinase gene; triangles, loxP sites; pA, polyadenylation signal. (B) Left panel: Southern blot analysis with the 5′external probe demonstrating recombination of the targeting construct (hpmneo-tk) into the wt β1 integrin locus giving rise to a 22.9 kb wt band and a 7 kb recombinant band. Right panel: Southern blot analysis with the 3′external probe demonstrating the deletion of the neo-tk cassette from the knockin hpmKIneo-tk+ allele (hpmKIlox) with a 22.9 kb wt band and 16 kb hpmKIlox band. (C) Representative PCR on genomic DNA from tail snips of offspring from hpmKIlox/+ intercrosses using a forward primer (primer S) binding to the transmembrane coding sequence of the β1 integrin gene, within exon 15 and a reverse primer (primer AS) binding to an intronic sequence downstream of exon 15. The 423 and 191 bp DNA bands correspond to the hpmKIlox and the wt β1 integrin allele, respectively.
(0.95 MB TIF)

Figure S2 Integrin profile of hpm keratinocytes. Cell surface expression of integrins in freshly isolated keratinocytes from control and hpm mice at indicated ages were assessed by flow cytometry. Keratinocytes were stained with antibodies against α2, β4 and α6 integrins. Expression of integrin subunits was normalized to the expression level of age-matched controls. Cell surface expression of α2 integrin is significantly reduced in 3-week-old hpm mice and increases to control levels in 20-month-old hpm mice. Expression of α6 and β4 integrin in young and old hpm mutant mice is increased compared to controls (middle and lower panel, respectively). Mean fluorescence intensities were corrected for background fluorescence. Error bars indicate s.d. At least 2 control and 3 hpm mutant mice per developmental stage were analysed.
(0.90 MB TIF)

Figure S3 Wound healing in hpm mice. (A) Morphometrical analysis of wound healing parameters of 5 day wounds. Slightly reduced wound closure in hpm mice (control: n = 19, N = 7; hpm: n = 21, N = 7). The area of the hyperproliferative epithelium (HE) was similar in 5 day wounds of control and hpm mice (control: n = 14, N = 6; hpm: n = 11, N = 6). (B) Morphometrical analysis of wound healing parameters 13 days after wounding. The wound width was significantly increased (p = 0.0005; control: n = 16, N = 5; hpm: n = 16, N = 5) and the epidermis still hyperthickened in 13 day wounds of hpm mice (p = 0.0013; control: n = 19, N = 7; hpm: n = 21, N = 7). (C) 13 day wounds were examined for lacZ expression. Black arrowheads indicate the edges of the wound, white arrowheads mark an area of lacZ negative keratinocytes in the middle of the wound epithelium (control: N = 3; hpm: N = 4). Boxed area is shown at higher magnification. n, number of measurements; N, number of mice.
(6.50 MB TIF)
