# Supplementary figures and images for: β1 Integrin-Mediated Adhesion Signalling Is Essential for Epidermal Progenitor Cell Expansion
Source: PLoS One. 2009 May 8;4(5):e5488. doi: 10.1371/journal.pone.0005488 (PMC2676508; doi:10.1371/journal.pone.0005488)

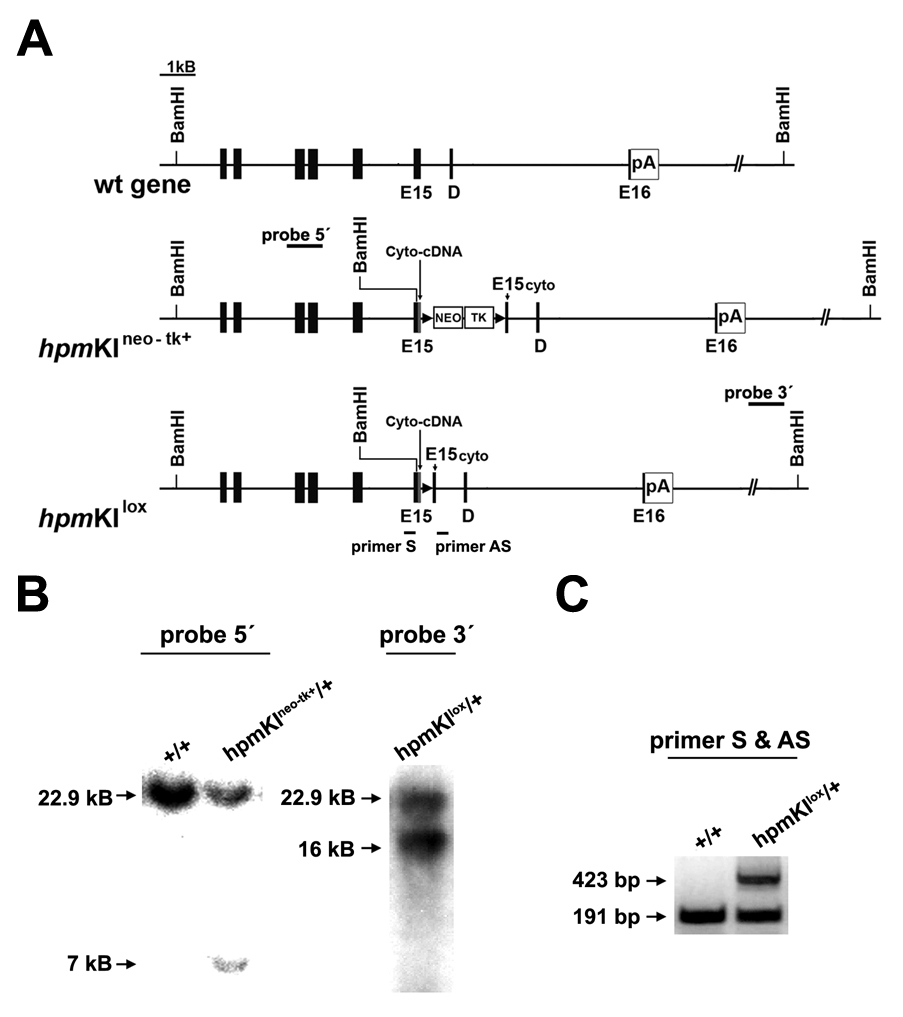

Supplement: Figure S1 — Targeting strategy for the hpm allele. (A) Partial map of the β1A wild-type (wt), the hpm knock-in allele before (hpmKIneo-tk+) and after Cre-mediated deletion of the floxed neo-tk cassette (hpmKIlox). External probes used for Southern blotting after BamHI digest and genotyping PCR primers are indicated. Cyto-cDNA, a wt 141 bp cDNA fragment coding for the complete cytoplasmic domain of the β1A integrin gene including the endogenous stop codon; E15cyto, endogenous sequence of exon 15 encoding for the membrane proximal part of the β1 integrin cytoplasmic tail; filled boxes, exons; D, exon D; neo, neomycin resistance gene; tk, thymidine kinase gene; triangles, loxP sites; pA, polyadenylation signal. (B) Left panel: Southern blot analysis with the 5′external probe demonstrating recombination of the targeting construct (hpmneo-tk) into the wt β1 integrin locus giving rise to a 22.9 kb wt band and a 7 kb recombinant band. Right panel: Southern blot analysis with the 3′external probe demonstrating the deletion of the neo-tk cassette from the knockin hpmKIneo-tk+ allele (hpmKIlox) with a 22.9 kb wt band and 16 kb hpmKIlox band. (C) Representative PCR on genomic DNA from tail snips of offspring from hpmKIlox/+ intercrosses using a forward primer (primer S) binding to the transmembrane coding sequence of the β1 integrin gene, within exon 15 and a reverse primer (primer AS) binding to an intronic sequence downstream of exon 15. The 423 and 191 bp DNA bands correspond to the hpmKIlox and the wt β1 integrin allele, respectively. (0.95 MB TIF) [file pone.0005488.s001.tif]

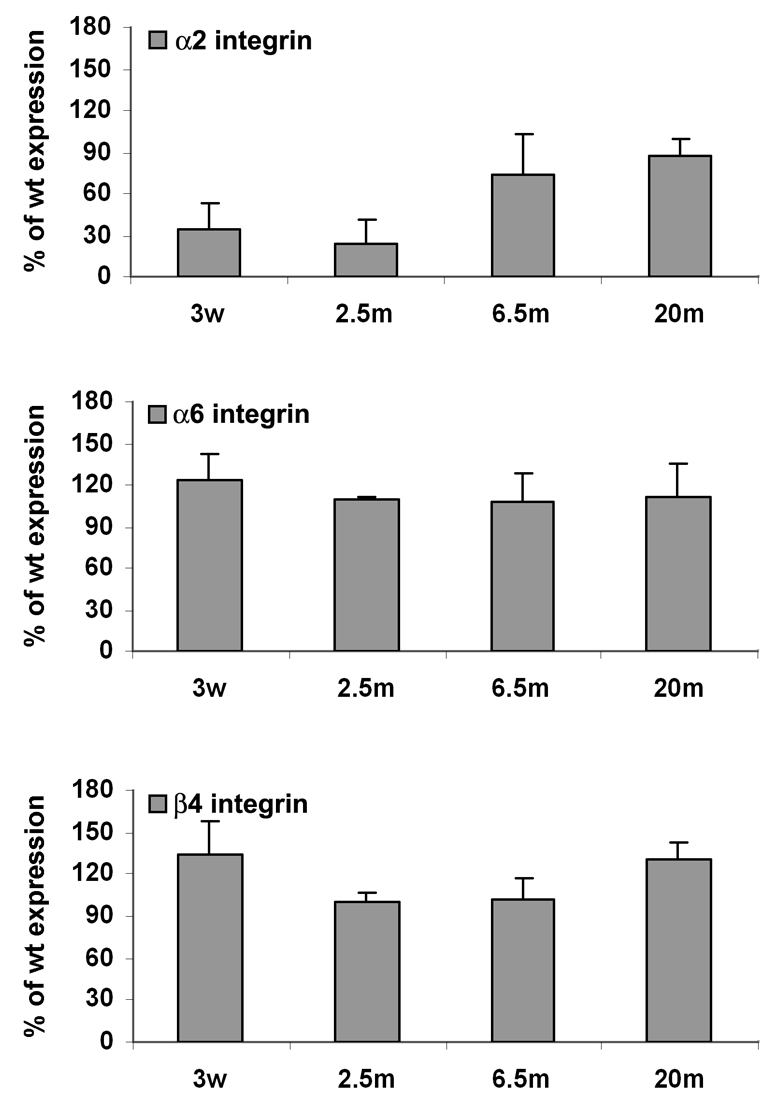

Supplement: Figure S2 — Integrin profile of hpm keratinocytes. Cell surface expression of integrins in freshly isolated keratinocytes from control and hpm mice at indicated ages were assessed by flow cytometry. Keratinocytes were stained with antibodies against α2, β4 and α6 integrins. Expression of integrin subunits was normalized to the expression level of age-matched controls. Cell surface expression of α2 integrin is significantly reduced in 3-week-old hpm mice and increases to control levels in 20-month-old hpm mice. Expression of α6 and β4 integrin in young and old hpm mutant mice is increased compared to controls (middle and lower panel, respectively). Mean fluorescence intensities were corrected for background fluorescence. Error bars indicate s.d. At least 2 control and 3 hpm mutant mice per developmental stage were analysed. (0.90 MB TIF) [file pone.0005488.s002.tif]

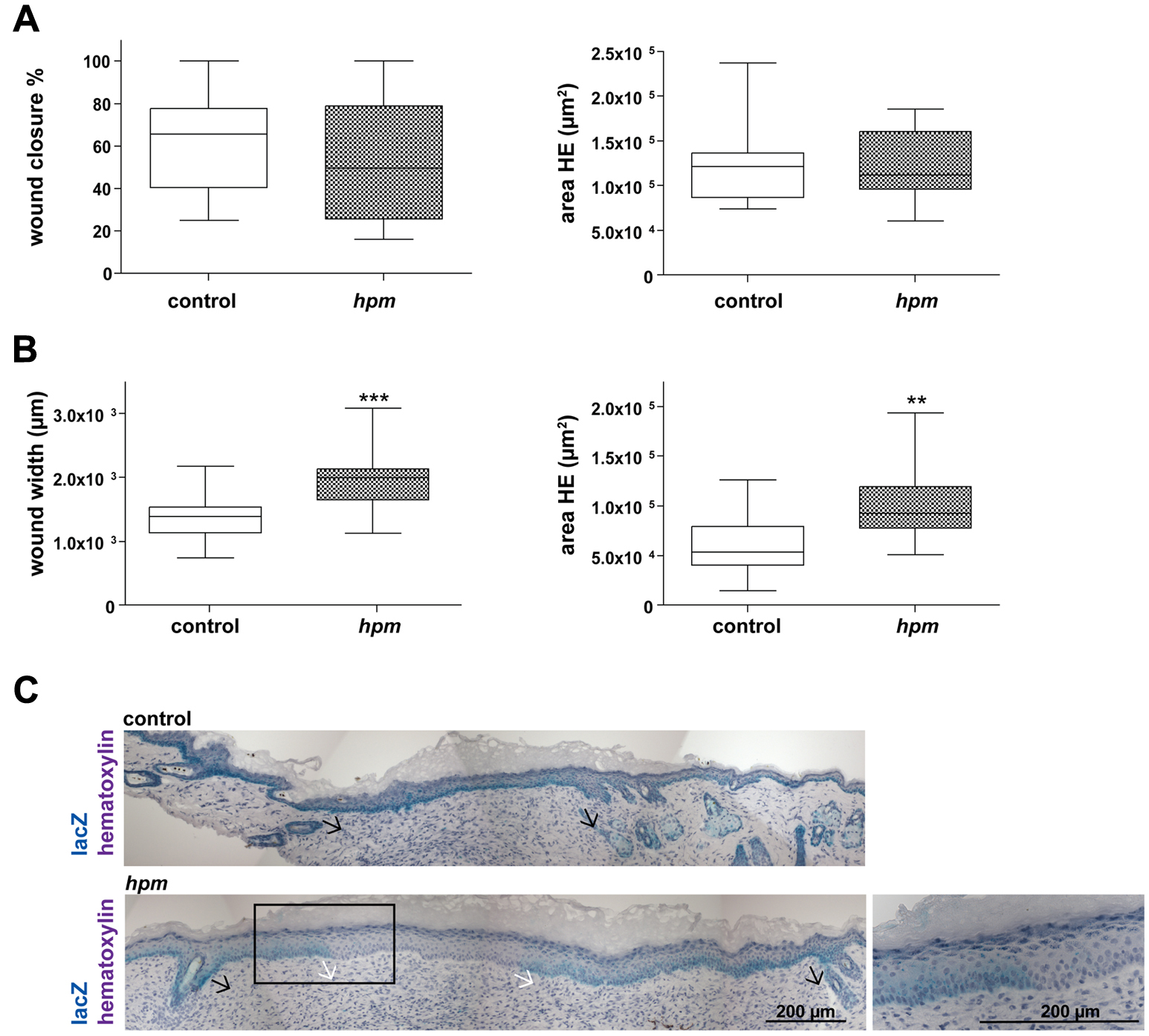

Supplement: Figure S3 — Wound healing in hpm mice. (A) Morphometrical analysis of wound healing parameters of 5 day wounds. Slightly reduced wound closure in hpm mice (control: n = 19, N = 7; hpm: n = 21, N = 7). The area of the hyperproliferative epithelium (HE) was similar in 5 day wounds of control and hpm mice (control: n = 14, N = 6; hpm: n = 11, N = 6). (B) Morphometrical analysis of wound healing parameters 13 days after wounding. The wound width was significantly increased (p = 0.0005; control: n = 16, N = 5; hpm: n = 16, N = 5) and the epidermis still hyperthickened in 13 day wounds of hpm mice (p = 0.0013; control: n = 19, N = 7; hpm: n = 21, N = 7). (C) 13 day wounds were examined for lacZ expression. Black arrowheads indicate the edges of the wound, white arrowheads mark an area of lacZ negative keratinocytes in the middle of the wound epithelium (control: N = 3; hpm: N = 4). Boxed area is shown at higher magnification. n, number of measurements; N, number of mice. (6.50 MB TIF) [file pone.0005488.s003.tif]
